# Supplementary figures and images for: Risk factors for Encapsulating Peritoneal Sclerosis in patients undergoing peritoneal dialysis: A meta-analysis
Source: PLoS One. 2022 Mar 21;17(3):e0265584. doi: 10.1371/journal.pone.0265584 (PMC8936465; doi:10.1371/journal.pone.0265584)

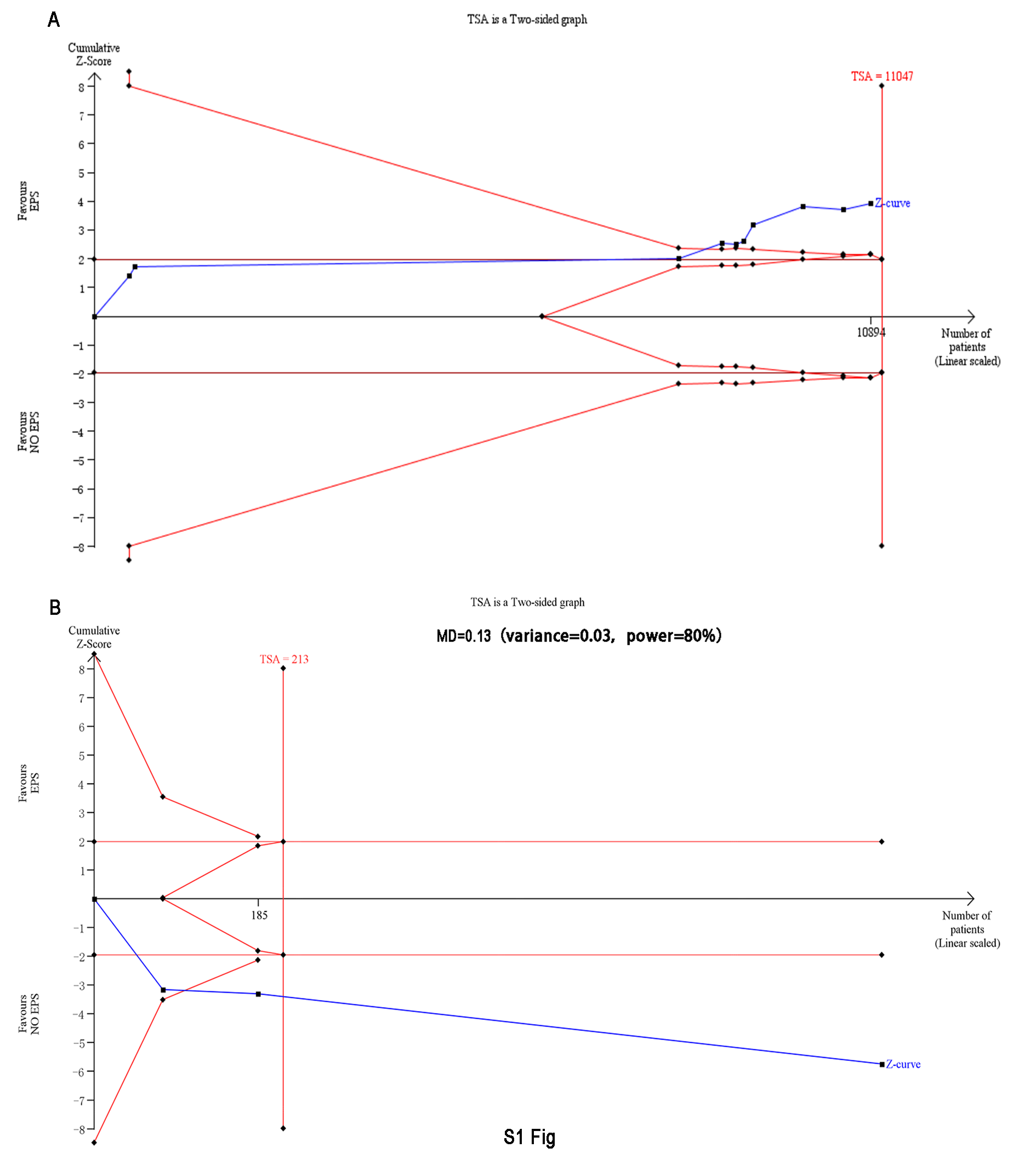

Supplement: S1 Fig — (A) Age at PD onset; (B) D/P Cr. The information size was calculated based on MD, alpha of 5%, power of 80%. (TIF) [file pone.0265584.s001.tif]

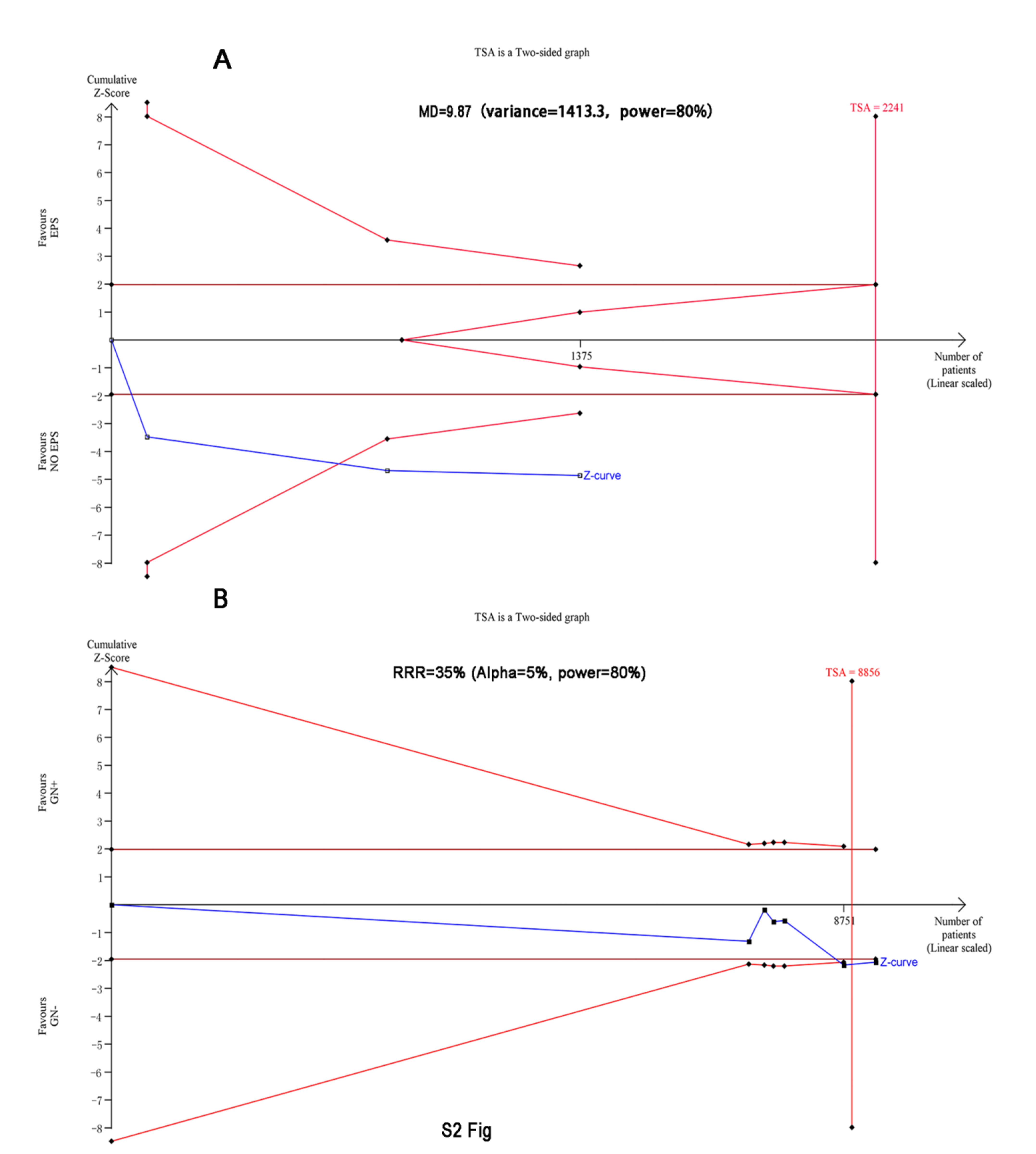

Supplement: S2 Fig — (A) Peritonitis duration; (B) History of GN. The information size was calculated based on relative risk reduction (RRR) or MD, alpha of 5%, power of 80%. (TIF) [file pone.0265584.s002.tif]

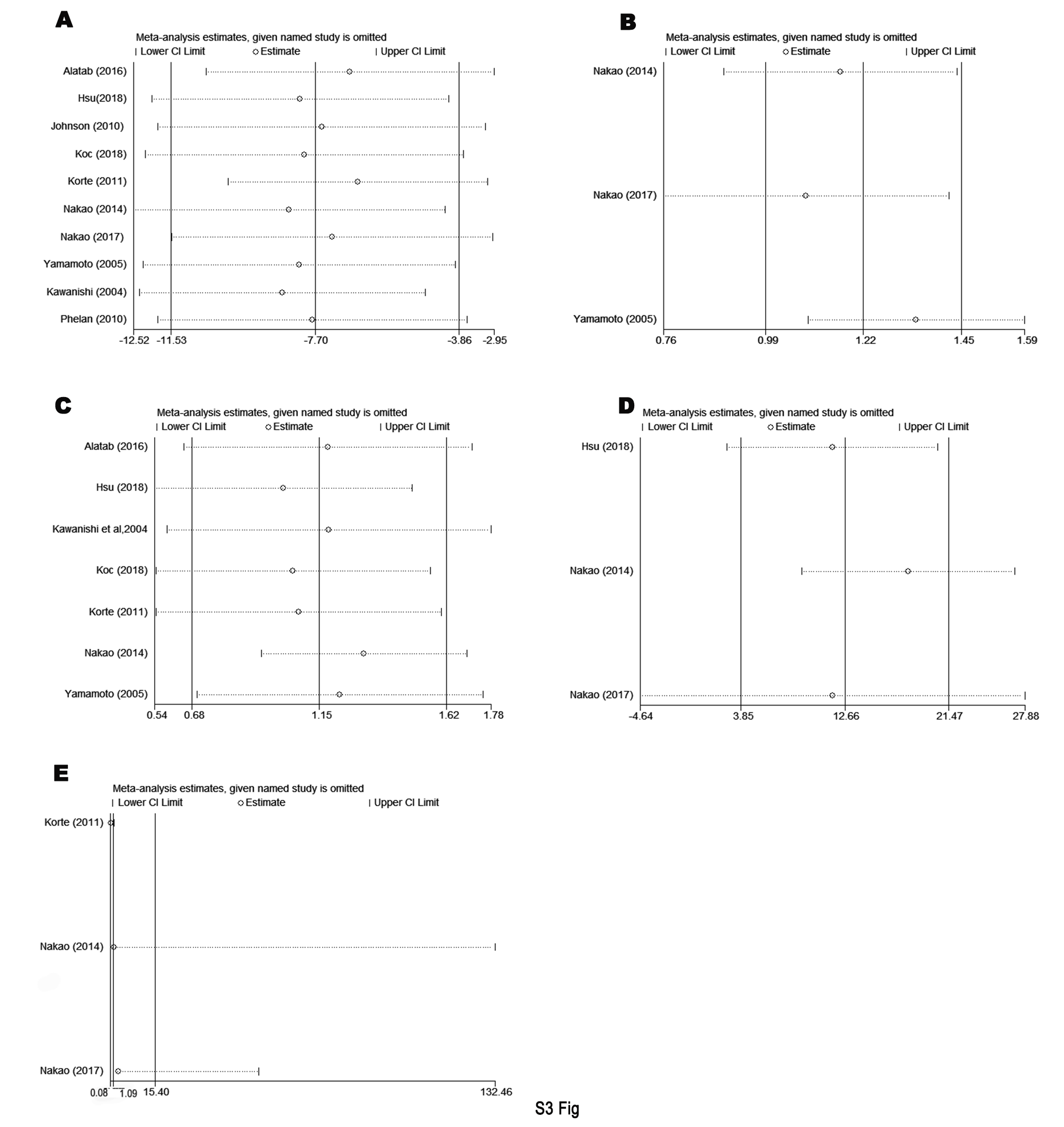

Supplement: S3 Fig — (A) Age on dialysis. (B) D/P Cr. (C) PD duration. (D) peritonitis duration. (E) Use of icodextrin. (TIF) [file pone.0265584.s003.tif]
