# Supplementary material for: Risk factors for Encapsulating Peritoneal Sclerosis in patients undergoing peritoneal dialysis: A meta-analysis
Source: PLoS One. 2022 Mar 21;17(3):e0265584. doi: 10.1371/journal.pone.0265584 (PMC8936465; doi:10.1371/journal.pone.0265584)
Supplement: S2 File — (DOCX) [file pone.0265584.s005.docx]

**S2 File: Search** **strategy:**

**January 1, 2022**

| **Database** | **Results** |
| --- | --- |
| **PubMed** | 86 |
| **Web of science** | 279 |
| **Embase** | 99 |
| **China Biology Medicine (CBM)** | 20 |
| **Cochrane Library** | 9 |
| **manually search** | 2 |

**Search strategy in PubMed:**

#1 "Peritoneal Fibrosis"[Mesh]

#2 Peritoneal Fibrosing Syndrome[Title/Abstract]

#3 Peritoneal Sclerosis[Title/Abstract]

#4 Encapsulating Peritoneal Sclerosis[Title/Abstract]

#5 Sclerosing Encapsulating Peritonitis[Title/Abstract]

#6 peritoneal thickening[Title/Abstract]

#7 sclerosing peritonitis [Title/Abstract]

#8 #1 OR #2 OR #3 OR #4 OR #5 OR #6 OR #7

#9 "Peritoneal Dialysis"[Mesh]

#10 renal dialysis[Title/Abstract]

#11 renal replacement therapy[Title/Abstract]

#12 chronic kidney disease[Title/Abstract]

#13 end-stage kidney disease[Title/Abstract]

#14 ESKD[Title/Abstract]

#15 #9 OR #10 OR #11 OR #12 OR #13 OR # 14

#16 #8 AND #15

#17 “Risk Factor”[MeSH]

#18 risk factors[Title/Abstract]

#19 influence factors[Title/Abstract]

#20 factor analysis[Title/Abstract]

#21 #17 OR #18 OR #19 OR #20

#22 #16 AND #21
