# Supplementary material for: Risk factors for Encapsulating Peritoneal Sclerosis in patients undergoing peritoneal dialysis: A meta-analysis
Source: PLoS One. 2022 Mar 21;17(3):e0265584. doi: 10.1371/journal.pone.0265584 (PMC8936465; doi:10.1371/journal.pone.0265584)
Supplement: S3 File — (PDF) [file pone.0265584.s006.pdf]

National Institute for Health Research

UNIVERSITY of York
Centre for Reviews and Dissemination

Systematic review

1. * Review title.

Give the title of the review in English

Risk factors for Encapsulating Peritoneal Sclerosis in patients undergoing peritoneal dialysis: a meta-analysis

2. Original language title.

For reviews in languages other than English, give the title in the original language. This will be displayed with the English language title.

3. * Anticipated or actual start date.

Give the date the systematic review started or is expected to start.

01/01/2022

4. * Anticipated completion date.

Give the date by which the review is expected to be completed.

10/02/2022

5. * Stage of review at time of this submission.

This field uses answers to initial screening questions. It cannot be edited until after registration.

Tick the boxes to show which review tasks have been started and which have been completed.

Update this field each time any amendments are made to a published record.

The review has not yet started: No

Review stage	Started	Completed
Preliminary searches	Yes	No
Piloting of the study selection process	No	No
Formal screening of search results against eligibility criteria	No	No
Data extraction	No	No
Risk of bias (quality) assessment	No	No
Data analysis	No	No

Provide any other relevant information about the stage of the review here.

6. * Named contact.

The named contact is the guarantor for the accuracy of the information in the register record. This may be any member of the review team.

Dashan Li

Email salutation (e.g. "Dr Smith" or "Joanne") for correspondence:

Dr Li

7. * Named contact email.

Give the electronic email address of the named contact.

lidashanyjs@163.com

8. Named contact address

Give the full institutional/organisational postal address for the named contact.

The First Affiliated Hospital of Anhui Medical University, Jixi Road, Hefei City, Anhui Province, China

9. Named contact phone number.

Give the telephone number for the named contact, including international dialling code.

17864391049

10. * Organisational affiliation of the review.

Full title of the organisational affiliations for this review and website address if available. This field may be completed as 'None' if the review is not affiliated to any organisation.

Anhui Medical University

Organisation web address:

11. * Review team members and their organisational affiliations.

Give the personal details and the organisational affiliations of each member of the review team. Affiliation refers to groups or organisations to which review team members belong. **NOTE: email and country now MUST be entered for each person, unless you are amending a published record.**

Dr Li Da shan. The First Affiliated Hospital of Anhui Medical University
Dr Li Yuanyuan. The First Affiliated Hospital of Anhui Medical University
Dr Zeng Hanxu. The First Affiliated Hospital of Anhui Medical University
Dr Wu Yonggui. The First Affiliated Hospital of Anhui Medical University

12. * Funding sources/sponsors.

Details of the individuals, organizations, groups, companies or other legal entities who have funded or sponsored the review.

None

Grant number(s)

State the funder, grant or award number and the date of award

13. * Conflicts of interest.

List actual or perceived conflicts of interest (financial or academic).

None

14. Collaborators.

Give the name and affiliation of any individuals or organisations who are working on the review but who are not listed as review team members. **NOTE: email and country must be completed for each person, unless you are amending a published record.**

15. * Review question.

State the review question(s) clearly and precisely. It may be appropriate to break very broad questions down into a series of related more specific questions. Questions may be framed or refined using PI(E)COS or similar where relevant.

1. What risk factors are significantly associated with EPS patients?

To systemically evaluate the risk factors association with Encapsulating Peritoneal Sclerosis(EPS) in peritoneal dialysis patients. The doctors can identify the risk factors of EPS, which is beneficial to reduce the incidence rate of EPS and prolong survival time .

16. * Searches.

State the sources that will be searched (e.g. Medline). Give the search dates, and any restrictions (e.g. language or publication date). Do NOT enter the full search strategy (it may be provided as a link or attachment below.)

We planed to searched relevant citations in PubMed, Embase, Web of Science, Cochrane Library, and China Biology Medicine (CBM) from their inception to January 1st, 2022, and the bibliographies from the citations of relevant articles were manually searched. We also searched the http://www.greylit.org/ website for studies that were registered as completed but not yet published. There were no restrictions on language with limited to human subjects.

17. URL to search strategy.

Upload a file with your search strategy, or an example of a search strategy for a specific database, (including the keywords) in pdf or word format. In doing so you are consenting to the file being made publicly accessible. Or provide a URL or link to the strategy. Do NOT provide links to your search **results**.

Alternatively, upload your search strategy to CRD in pdf format. Please note that by doing so you are consenting to the file being made publicly accessible.

Yes I give permission for this file to be made publicly available

18. * Condition or domain being studied.

Give a short description of the disease, condition or healthcare domain being studied in your systematic review.

Encapsulating Peritoneal Sclerosis (EPS), also known as Peritoneal Fibrosis and Sclerosing Encapsulating Peritonitis, is a rare but the most severe complication associated with peritoneal dialysis, especially in patients who are on long-term PD. In 2000, the International Society for Peritoneal Dialysis has described EPS as a rare clinical syndrome characterized by intermittent, persistent or recurrent intestinal obstruction caused by diffuse adhesions of a thickened and sclerosing peritoneum. Adverse outcomes of PD-related EPS are diverse, including loss of incipient peritoneal ultrafiltration capacity, a higher transport status, and a low residual kidney function. The overall incidence of EPS obtained from a variety of countries is low and varies between 0.5% and 7.3%. A registry study from Australian reported that the incidence rate increased with duration of PD therapy . Similar results were obtained in a prospective study from Japan, in which the incidence of EPS was 0.7%, 2.1%, 5.9%, 17.2% in patients who had been on PD for more than 5, 8, 10, and 15 years, respectively.This review was conducted to identify comprehensive results which could contribute to develop clinical strategies for early prevention of EPS

19. * Participants/population.

Specify the participants or populations being studied in the review. The preferred format includes details of both inclusion and exclusion criteria.

The exposure or case group was the participants with Encapsulating Peritoneal Sclerosis.

20. * Intervention(s), exposure(s).

Give full and clear descriptions or definitions of the interventions or the exposures to be reviewed. The preferred format includes details of both inclusion and exclusion criteria.

Intervention or exposure was any risk factor association with Encapsulating Peritoneal Sclerosis.

21. * Comparator(s)/control.

Where relevant, give details of the alternatives against which the intervention/exposure will be compared (e.g. another intervention or a non-exposed control group). The preferred format includes details of both inclusion and exclusion criteria.

The control group was the peritoneal dialysis patients without Encapsulating Peritoneal Sclerosis.

22. * Types of study to be included.

Give details of the study designs (e.g. RCT) that are eligible for inclusion in the review. The preferred format includes both inclusion and exclusion criteria. If there are no restrictions on the types of study, this should be

stated.

This review included case-control studies, cross-sectional studies cohort studies, and randomized controlled trials (RCTs) to explore the risk factors associated with Encapsulating Peritoneal Sclerosis. The studies were conducted in patients whose peritoneal dialysis lasted three months at least and age 18years.

23. Context.

Give summary details of the setting or other relevant characteristics, which help define the inclusion or exclusion criteria.

24. * Main outcome(s).

Give the pre-specified main (most important) outcomes of the review, including details of how the outcome is defined and measured and when these measurement are made, if these are part of the review inclusion criteria.

The risk factors associated with Encapsulating Peritoneal Sclerosis.

The data will be extracted to generate the odds ratio or mean difference(with confidence limits) for effect of the risk factors.

Measures of effect

Please specify the effect measure(s) for you main outcome(s) e.g. relative risks, odds ratios, risk difference, and/or 'number needed to treat.

Variables with different units will be converted to a unanimous unit.

The data was presented by odds ratios (OR) or mean difference(MD) with their 95% confidence intervals (CIs).

25. * Additional outcome(s).

List the pre-specified additional outcomes of the review, with a similar level of detail to that required for main outcomes. Where there are no additional outcomes please state ‘None’ or ‘Not applicable’ as appropriate to the review

Not applicable

Measures of effect

Please specify the effect measure(s) for you additional outcome(s) e.g. relative risks, odds ratios, risk difference, and/or 'number needed to treat.

None

26. * Data extraction (selection and coding).

Describe how studies will be selected for inclusion. State what data will be extracted or obtained. State how this will be done and recorded.

Two authors searched and extracted the data of the included primary studies, aggregating the resulting data into a structured table: first author's name, year of publication, study type, country/region, sample size, and incidence. Any discrepancies regarding study selection and data extraction was resolved through consensus and arbitrated by the third author if necessary.

27. * Risk of bias (quality) assessment.

State which characteristics of the studies will be assessed and/or any formal risk of bias/quality assessment tools that will be used.

The ROBINS-I (Risk of Bias in Non-randomized studies of Interventions) tool was used to evaluate the risk of bias of included studies based on the following seven criteria: bias due to confounding; bias due to selection of participants; bias in classification of interventions; bias due to deviations from intended interventions; bias due to missing data; bias in measurement of outcomes; bias in selection of the reported result. This tool categorizes the risk of bias as low, moderate, serious, critical, and unclear. Disagreements were resolved by consultation with a third reviewer.

28. * Strategy for data synthesis.

Describe the methods you plan to use to synthesise data. This **must not be generic text** but should be **specific to your review** and describe how the proposed approach will be applied to your data. If meta-analysis is planned, describe the models to be used, methods to explore statistical heterogeneity, and software package to be used.

All statistics were collated and analyzed using the Review Manager 5.3 and Stata 14.0 software. Continuous variables were summarized using mean difference (MD) or standardized mean difference (SMD) with their 95% confidence intervals (CI), For dichotomous variables, odds ratio (OR) with their 95% CI were estimated. In addition, heterogeneity was quantified using the Q test and I^2 statistics. When I^2 was 50% and Q chisquared test result 0.1, it shows that there in no large heterogeneity among the trials and the fixed-effect model was used; else a random-effect model was used. A trial sequence analysis (TSA) was used to analyze the sample size required for this meta-analysis to improve the credibility of this study.

29. * Analysis of subgroups or subsets.

State any planned investigation of ‘subgroups’. Be clear and specific about which type of study or participant will be included in each group or covariate investigated. State the planned analytic approach.

When large heterogeneity was present, sensitivity analysis were performed to identify responsible outlier studies. We planned to conduct subgroup analyses for different kind of outcome data or characteristics of risk factors if data were available.

30. * Type and method of review.

Select the type of review, review method and health area from the lists below.

Type of review

Cost effectiveness
No

Diagnostic
No

Epidemiologic

No

Individual patient data (IPD) meta-analysis
No

Intervention
No

Living systematic review
No

Meta-analysis
Yes

Methodology
No

Narrative synthesis
No

Network meta-analysis
No

Pre-clinical
No

Prevention
Yes

Prognostic
No

Prospective meta-analysis (PMA)
No

Review of reviews
No

Service delivery
No

Synthesis of qualitative studies
No

Systematic review
Yes

Other
No

Health area of the review

Alcohol/substance misuse/abuse
No

Blood and immune system
No

Cancer
No

Cardiovascular
No

Care of the elderly
No

Child health
No

Complementary therapies
No

COVID-19
No

Crime and justice
No

Dental
No

Digestive system
No

Ear, nose and throat
No

Education
No

Endocrine and metabolic disorders
No

Eye disorders
No

General interest
No

Genetics
No

Health inequalities/health equity
No

Infections and infestations
No

International development
No

Mental health and behavioural conditions
No

Musculoskeletal
No

Neurological
No

Nursing
No

Obstetrics and gynaecology
No

Oral health

No

Palliative care
No

Perioperative care
No

Physiotherapy
No

Pregnancy and childbirth
No

Public health (including social determinants of health)
No

Rehabilitation
No

Respiratory disorders
No

Service delivery
No

Skin disorders
No

Social care
No

Surgery
No

Tropical Medicine
No

Urological
Yes

Wounds, injuries and accidents
No

Violence and abuse
No

31. Language.

Select each language individually to add it to the list below, use the bin icon to remove any added in error.
English

There is an English language summary.

32. * Country.

Select the country in which the review is being carried out. For multi-national collaborations select all the countries involved.

China

33. Other registration details.

Name any other organisation where the systematic review title or protocol is registered (e.g. Campbell, or The Joanna Briggs Institute) together with any unique identification number assigned by them. If extracted data will be stored and made available through a repository such as the Systematic Review Data Repository (SRDR), details and a link should be included here. If none, leave blank.

34. Reference and/or URL for published protocol.

If the protocol for this review is published provide details (authors, title and journal details, preferably in Vancouver format)

Add web link to the published protocol.

Or, upload your published protocol here in pdf format. Note that the upload will be publicly accessible.

Yes I give permission for this file to be made publicly available

Please note that the information required in the PROSPERO registration form must be completed in full even if access to a protocol is given.

35. Dissemination plans.

Do you intend to publish the review on completion?

Yes

Give brief details of plans for communicating review findings.?

36. Keywords.

Give words or phrases that best describe the review. Separate keywords with a semicolon or new line. Keywords help PROSPERO users find your review (keywords do not appear in the public record but are included in searches). Be as specific and precise as possible. Avoid acronyms and abbreviations unless these are in wide use.

37. Details of any existing review of the same topic by the same authors.

If you are registering an update of an existing review give details of the earlier versions and include a full bibliographic reference, if available.

38. * Current review status.

Update review status when the review is completed and when it is published.New registrations must be ongoing so this field is not editable for initial submission.
Please provide anticipated publication date

Review_Ongoing

39. Any additional information.

Provide any other information relevant to the registration of this review.

40. Details of final report/publication(s) or preprints if available.

Leave empty until publication details are available OR you have a link to a preprint (NOTE: this field is not editable for initial submission). List authors, title and journal details preferably in Vancouver format.

Give the link to the published review or preprint.
