# Supplementary material for: Risk factors for Encapsulating Peritoneal Sclerosis in patients undergoing peritoneal dialysis: A meta-analysis
Source: PLoS One. 2022 Mar 21;17(3):e0265584. doi: 10.1371/journal.pone.0265584 (PMC8936465; doi:10.1371/journal.pone.0265584)
Supplement: S1 Table — (DOCX) [file pone.0265584.s007.docx]

**S1 Table. Quality assessment of the included studies using ROBINS-I tool.**

| Study | Bias due to confounding | Bias in selection of participants into the study | Bias in classification of interventions | Bias due to deviations from intended interventions | Bias due to missing data | Bias in measurement of outcomes | Bias in selection of the reported result | Overall |
| --- | --- | --- | --- | --- | --- | --- | --- | --- |
| Alatab et al. | Moderate | Low | Moderate | Low | Low | Low | Low | Moderate |
| Nakao et al.(2014) | Moderate | Low | Low | Low | Moderate | Low | Low | Moderate |
| Nakao et al. (2017) | Moderate | Low | Low | Low | Low | Low | Low | Moderate |
| Yamamoto et al. | Moderate | Low | Low | Low | Low | Low | Low | Moderate |
| Korte et al. | Moderate | Low | Low | Low | Low | Low | Low | Moderate |
| Hsu et al. | Moderate | Low | Moderate | Low | Low | Low | Low | Moderate |
| Johnson et al. | Moderate | Low | Low | Low | Low | Low | Low | Moderate |
| Koc et al. | Moderate | Low | Low | Low | Moderate | Low | Low | Moderate |
| Kawanishi et al. | Moderate | Low | Low | Low | Low | Low | Low | Moderate |
| Phelan et al. | Moderate | Low | Low | Moderate | Low | Low | Low | Moderate |
