# Supplementary material for: Risk factors for Encapsulating Peritoneal Sclerosis in patients undergoing peritoneal dialysis: A meta-analysis
Source: PLoS One. 2022 Mar 21;17(3):e0265584. doi: 10.1371/journal.pone.0265584 (PMC8936465; doi:10.1371/journal.pone.0265584)
Supplement: S2 Table — (DOCX) [file pone.0265584.s008.docx]

**S2 Table. Publication bias associated with potential risk factors for EPS.**

| **Risk factors** | **Number of studies** | ***P*** |
| --- | --- | --- |
| Age on dialysis | 10 | 0.640 |
| Sex | 9 | 0.205 |
| D/P Cr^a^ | 3 | 0.284 |
| PD duration | 7 | 0.711 |
| Peritonitis duration | 3 | 0.316 |
| Peritonitis history | 3 | 0.188 |
| Use of icodextrin | 3 | 0.166 |
| History of glomerulonephritis | 6 | 0.387 |
| History of polycystic kidney disease | 6 | 0.467 |

^a^ D/P C, dialysate-to-plasma creative ratio
